# Plasma neutrophil gelatinase associated lipocalin (NGAL) is associated with kidney function in uraemic patients before and after kidney transplantation

**DOI:** 10.1186/1471-2369-13-8

**Published:** 2012-02-10

**Authors:** Nils E Magnusson, Mads Hornum, Kaj Anker Jørgensen, Jesper Melchior Hansen, Claus Bistrup, Bo Feldt-Rasmussen, Allan Flyvbjerg

**Affiliations:** 1 The Medical Research Laboratories, Faculty of Health Sciences, Aarhus University, Aarhus, Denmark; 2 Department of Endocrinology and Internal Medicine, Aarhus University Hospital, Aarhus, Denmark; 3 Department of Nephrology, Copenhagen University Hospital, Rigshospitalet, Copenhagen, Denmark; 4 Department of Renal Medicine C, Aarhus University Hospital, Skejby, Denmark; 5 Department of Nephrology, Copenhagen University Hospital,Herlev Hospital, Copenhagen, Denmark; 6 Department of Nephrology, Odense University Hospital, Odense, Denmark

## Abstract

**Background:**

Neutrophil gelatinase associated lipocalin (NGAL) is a biomarker of kidney injury. We examined plasma levels of NGAL in a cohort of 57 kidney allograft recipients (Tx group, 39 ± 13 years), a uraemic group of 40 patients remaining on the waiting list (47 ± 11 years) and a control group of 14 healthy subjects matched for age, sex and body mass index (BMI). The kidney graft recipients were studied at baseline before transplantation and 3 and 12 months after transplantation and the uraemic group at baseline and after 12 months.

**Methods:**

NGAL was measured using a validated in-house Time-Resolved Immuno-flourometric assay (TRIFMA). Repeated measurements differed by < 10% and mean values were used for statistical analyses. Spearman rank order correlation analysis and the Kruskal-Wallis non-parametric test were used to evaluate the association of NGAL concentrations with clinical parameters.

**Results:**

Plasma NGAL levels before transplantation in the Tx and uraemic groups were significantly higher than in the healthy controls (1,251 μg/L, 1,478 μg/L vs. 163 μg/L, p < 0.0001). In the Tx group NGAL concentrations were associated with serum creatinine (R = 0.51, p < 0.0001), duration of end-stage renal failure (R = 0.41, p = 0.002) and leukocyte count (R = 0.29, p < 0.026). At 3 and 12 months plasma NGAL concentrations declined to 223 μg/L and 243 μg/L, respectively and were associated with homocysteine (R = 0.39, p = 0.0051 and R = 0.47, p = 0.0007).

**Conclusions:**

Plasma NGAL is a novel marker of kidney function, which correlates to duration of end-stage renal failure (ESRD) and serum creatinine in uraemic patients awaiting kidney transplantation. Plasma NGAL is associated with homocysteine in transplanted patients. The prognostic value of these findings requires further studies.

## Background

Neutrophil gelatinase associated lipocalin (NGAL) also known as Lipocalin 2 or Lcn2 is a 25 kDa protein identified originally as a protein associated with matrix metalloproteinase 9 (MMP-9) of human neutrophils [1]. Lipocalins are extracellular proteins which share a common tertiary structure that forms a barrel-like hydrophobic ligand binding site [2]. When bound to MMP-9, NGAL protects it from proteolytic degradation sustaining the proteolytic activity of MMP-9. No specific receptor for NGAL has yet been identified. However, the endocytosis low density lipoprotein receptor Megalin has been shown to bind NGAL with high affinity suggesting that NGAL is taken up by host cells [3]. NGAL has been suggested as a bacteriostatic agent indicating involvement of NGAL in the innate immune response [4-7]. Moreover, serum levels of NGAL have been reported to be useful in discriminating between acute bacterial and viral infections [8]. Expression of NGAL is prominent in secondary granules of human neutrophils [9,10]. NGAL is induced by bacterial lipopolysaccharides [11], dexamethasone [12,13], growth factors and cytokines such as insulin-like growth factor I (IGF-I) [12,14] and interleukin 1 (IL-1) [15-18]. Upon nephrotoxic and/or ischemic injury NGAL levels are highly increased in kidney cortical tubules, blood and urine [18]. In vascular smooth muscle cells NGAL expression is induced in response to vascular injury and depends on nuclear factor kappa B (NF-kB) expression [19]. Interestingly, the interaction of NGAL with MMP-9 may be a mechanism by which the proteolytic activity of MMP-9 is modulated in the vascular repair process indicating a role for NGAL in cardiovascular disease (CVD). Expression of NGAL is induced upon activation of Toll-like receptors (TLRs) on immune cells constituting an acute phase response to infection [20]. NGAL has received considerable attention due to its role as an early biomarker in kidney disease [21-23]. In a cross-sectional study of 100 kidney allograft recipients serum NGAL was shown to correlate with kidney function [24] and it has been studied in several clinical settings of acute kidney injury **(**AKI) [25-30]. Haase et al. [26] confirmed the predictive and prognostic value of NGAL as an early biomarker for AKI in a meta-analysis involving 19 studies ( > 2,500 patients). Induction of NGAL after kidney injury precedes the elevation of classical markers for kidney damage [27,28], e.g. serum creatinine, urinary N-acetyl glucosamidase and β2-microglobulin levels. In studies of renal failure in mice the functional significance of up-regulation of NGAL has been suggested to be renal protective [18,31]. The present study aimed to investigate the possible relationship between plasma NGAL levels and clinical parameters in a prospective study of non-diabetic uraemic patients.

## Methods

### Study procedure

The study population consisted of 97 non-diabetic uraemic patients and was based on a prospective, observational, national multicenter study. Data on the study population has been published previously [32]. In brief, 57 of the patients were scheduled for living donor kidney transplantation in the period between January 2006 and March 2008 at the four Danish transplantation centres: Rigshospitalet, Skejby, Odense, and Herlev University Hospitals (Tx group, age 39 ± 13 years). A control group of 40 patients from the transplantation waiting list at Rigshospitalet and Herlev University Hospitals (uraemic group, age 47 ± 11) was included. The study also included a group of healthy controls (n = 14). The healthy controls were BMI and sex matched and was recruited from public announcing. The regional ethical committee (# KF 01279825) and The Data Protection Agency (#2006-41-5640) approved the study. Participants gave their informed written consent. Transplanted participants were examined before transplantation and 3 and 12 months after transplantation. The uraemic controls were examined at baseline and 12 months later. The examinations were done after an overnight fast including coffee, tobacco, and exercise absence for 10 hours. Anti-hypertensive medication was allowed in the morning. A 75-gram oral glucose tolerance test (OGTT) was performed on all patients according to the WHO/ADA 2007 criteria [33]. An insulin sensitivity index was calculated according to Matsuda et al. [34]. Immunosuppression varied to some extent between the centers. Induction therapy included basiliximab (Simulect; Novartis), daclizumab (Zenapax; Roche), or antithymocyte globulin (Thymoglobulin; Genzyme B.V.). The majority of patients received 100-500 mg of intravenous methylprednisolone preoperatively, and treatment with oral prednisolone was started by 20-100 mg/d and was tapered to a dose of 7.5-10 mg at 3 months and to 5-7.5 mg at 9-12 months. Rejection episodes, indicated by increased plasma creatinine of 20% or greater for 2 days, or biopsy proven, were treated with intravenous methylprednisolone, 500 mg, for 3-5 days. For each patient the accumulated corticosteroid dose within the first 90 days was calculated and given in equivalents of prednisolone dose in grams. Forty-three patients started on cyclosporine (Sandimune Neoral; Novartis) at a dose of 2.5-6 mg/kg twice daily tapered to a trough level of whole-blood concentration of 150-300 μg/L for the first 3 months and 100-150 μg/L thereafter. The remaining 14 patients started on tacrolimus (Prograf; Astellas) at a dose of 0.075-0.15 mg/kg twice daily tapered to a whole-blood concentration of 8-15 μg/L for the first 3 months and 5-10 μg/L thereafter. A few patients (n = 8) changed from one treatment modality to another during the course of the study, and two patients were changed to rapamycin (Rapamune; Wyeth) treatment.

After 10 minutes of rest in the supine resting position arterial blood pressure (BP) was measured in triplicate from the arm opposite to a fistula or dialysis catheter. Mean arterial BP (MAP) was calculated on the basis of these three measurements. Renal function was estimated by the Cockcroft-Gault formula (eGFR) [35]. Fasting blood samples were drawn from an antecubital vein and aliquots of EDTA-plasma were stored at -80°C. Examiners were blinded with regards to the clinical and metabolic status of patients and clinical information was analyzed and described after completion of the data collection. Anti-hypertensive treatment mainly included β-blockade, calcium channel blockade, angiotensin-II-blockade and diuretics. Anti-hypertensive medication was stopped at the time of transplantation and thereafter titrated aiming a BP below 130/80 mmHg. Clinical characteristics of the study group are shown in Table 1.

**Table 1:** Demographic and clinical data, from 57 kidney allograft recipients (Tx group), 40 uraemic patients (uraemic controls) and 14 healthy controls, at baseline before transplantation.

	Tx-group	Uraemic group	Healthy controls
	N = 57	N = 40	N = 14
Age (years, SD)	39 ± 13	47 ± 11	39 ± 11
Gender (male/female)	38/19	27/13	5-Sep
ESRD duration (months, range)	24 (0-134)	45 (1-168)	-
BMI (kg/m^2^)	24 ± 4	24 ± 4	24 ± 3
Waist-hip ratio	0.92 ± 0.09	0.91 ± 0.08	0.81 ± 0.08***
Ever Smoking, n (%)	27 (47)	26 (65)	9 (64)

**Diagnoses n (%)**			
Glomerulonefritis	28 (49)	13 (33)	-
Hypertensive	5 (9)	11 (28)	-
Kidney disease			
Vasculitis	1 (2)	1 (3)	-
PKD	3 (5)	7 (18)	-
Other/unknown	20 (35)	8 (20)	-
Apoplexi	3 (5)	3 (8)	-
Former transplanted	11 (19)	10 (25)	-

**Dialysis-status**			
HD	28 (49)	28 (70)	-
CAPD	19 (33)	9 (27)	-
Pre-dialysis	10 (18)	3 (8)	-
First degree relatives with DM	2 (4)	4 (10)	2 (14)
Fasting p-glucose (mmol/L)	5.1 ± 0.5	5.1 ± 0.5	5.0 ± 0.3
P-glucose at 2-hours (mmol/L)	7.4 ± 1	7.5 ± 2	5.4 ± 1***
HbA1c (%)	5.2 ± 0.4	5.2 ± 0.4	5.2 ± 0.2
Insulin sensitivity index	6.8 ± 4.0	7.9 ± 5.1	14.7 ± 7.0***
Insulin secretion index	36.9 ± 18.5	31.4 ± 17.3	27 ± 14
Systolic BP (mmHg)	142 ± 21	141 ± 24	118 ± 10***
Diastolic BP (mmHg)	85 ± 13	83 ± 14	73 ± 8***
Pulse	69 ± 11	72 ± 11	61 ± 10***

Data are presented as mean ± SD or median and range, numbers (n) and percent (%). HD: hemodialysis, CAPD: continuous ambulatory peritoneal dialysis, ESRD: end stage renal disease, Tx: transplantation, PKD: polycystic kidney disease, DM: diabetes mellitus, BP: blood pressure. Wilcoxon Rank Sum Test or two sample t-test used where appropriate to test: uraemic patients vs. Healthy controls; *p < 0.05, **p < 0.005, ***p < 0.0005.

### Statistical methods

Data analyses using clinical data were performed using Statistical Analysis Software (SAS^®^) version 9.1. Unless specified otherwise, continuous data is described as mean ± SD for normal distributions, and median and range for skewed distributions. Paired data within groups were compared by t-tests for normally distributed data, and group comparisons of continuous data were performed using two-sample t-test for normally distributed data and non-parametric Wilcoxon Rank Sum Test for not-normal distributed data. Chi squared or Fisher's exact tests were used for group comparisons between categorical data. Correlation analysis was done using Spearman Rank Analysis. Scatter plot, regression analysis and difference vs. average plots were performed using STATA version 11.1. P-values below 0.05 for parametric and non-parametric tests were considered significant.

### NGAL assay procedure

Plasma NGAL levels were determined using stored EDTA-plasma by an in-house time resolved immunofluorometric (TRIFMA) sandwich-type assay based on anti-NGAL antibodies and recombinant NGAL from R&D Systems (Abingdon, UK). Microtitre plates (Nunc, Roskilde, Denmark) were coated overnight at 4°C with monoclonal capture NGAL antibody dissolved in phosphate buffered saline (PBS). Plates were blocked for 2 hours at room temperature with assay buffer (1% (w/v) BSA and 0.05% (v/v) Tween 20 in PBS) and washed three times with 0.05% (v/v) Tween 20 in PBS (PBST). Standards were prepared using recombinant NGAL by serial dilutions of up to three decades. A pool of plasma from five healthy adult donors and plasma pool spiked with recombinant NGAL were included as internal controls. Between each step of the assay the plates were washed three times with PBST. Samples and controls were diluted 1:200 in assay buffer and incubated overnight at 4°C. Plates were then incubated with biotinylated monoclonal NGAL antibody in assay buffer and then incubated with streptavidin-europium (Perkin Elmer Life Sciences, Finland). Enhancement solution (Perkin Elmer Life Sciences) was added and plates were measured in a time-resolved fluorometer. Standards, samples and non-specific binding were analysed in duplicate. Non-specific background (NSB) averaged 5000 counts per second (cps), the lowest standard 8000 cps and the highest standard 1.4 × 10E06 cps. The detection limit was estimated at < 0.1 μg/L based on NSB + 3 SD. The coefficient of variation (CV) within assays of standards controls and unknown samples averaged less than 7% based on repetitive runs of identical plasma samples. The CV between assays was determined by repetitive analysis of standards and control samples. After 15 different runs the between assay CVs averaged < 12%. Repetitive freezing and thawing of plasma from the healthy donors was tested every other cycle up to eleven cycles and did not alter the levels of NGAL significantly (data not shown) in line with previously published data [36]. The linearity of the assay, estimated with plasma samples using serial dilutions up to 1:800 indicated a high precision over a wide range of concentrations (mean CV = 4.4%, range; 0.5-9%). Minor increases of NGAL levels was found with increasing dilutions in samples with high levels of NGAL ( > 1400 μg/L, CV = 8%).

### Recovery of recombinant NGAL

Recovery was based on plasma samples containing high, medium or low levels of NGAL. These samples were spiked with different amounts of exogenously added recombinant NGAL and diluted 1:200 to a final concentration of 3, 9 or 30 μg/L of recombinant NGAL. Samples without added NGAL were used as controls. The assay was analyzed with six replicates and repeated twice. For each sample the recovery was estimated as the mean percentage of the measured concentrations compared to the expected concentrations.

### Comparison between platforms

The in-house NGAL assay was compared back to back with a commercially available ELISA kit (036, Bioporto Diagnostics, Gentofte, Denmark) with samples of plasma and serum; 34 uraemic plasma samples and a collection of 33 serum samples were run on both platforms. The ELISA assay was recently validated [36]. The ELISA kit analysis was carried out as suggested by the manufacturer. Samples used for the in-house NGAL assay were prepared and diluted 1:200 as described above. The assays were performed on the same day to minimize day to day variation.

## Results

The relationship between plasma NGAL and clinical characteristics

NGAL data were not normally distributed and Spearman rank order correlation analysis and the Kruskal-Wallis non-parametric test were used to evaluate the association of NGAL concentrations with clinical parameters. In the Tx group mean NGAL levels were 1,251 ± 438 μg/L prior to Tx (time = 0) vs. 223 ± 110 μg/L (p < 0.001) and 243 ± 86 μg/L (p < 0.001) at 3 and 12 months post Tx, respectively, compared to 163 ± 29 μg/L in the healthy controls (Tx-group at time = 0 vs. healthy, p < 0.0005). Mean NGAL levels are shown in Table 2. Following transplantation the NGAL levels were significantly reduced, but remained above the levels found in the healthy controls (243 ± 86 μg/L vs. 163 ± 29 μg/L, p < 0.0001). NGAL levels in the uraemic group remaining on the waiting list were significantly higher than in the Tx group at baseline before transplantation (1,478 ± 403 μg/L vs. 1,251 ± 438 μ/L, p < 0.05) and may probably be explained by a significant higher mean age and duration of ESRD in the uraemic group (47 vs. 39 years, and 45 vs. 24 months) and hence differences in serum creatinine. There was no significant difference in NGAL levels between the groups when stratifying for OGTT status, immunosuppression treatment or age in the Tx group (data not shown).

**Table 2:** Kidney allograft recipient patients vs. waiting list patients and within groups *p < 0.05, ***p < 0.0005, all patients vs. healthy controls ^§§§^p < 0.0005 (students paired t-test).

	Tx group	Waiting list	Healthy
NGAL μg/L Time = 0	1,251 ± 438	1,478 ± 403*	163 ± 29^§§§^

NGAL μg/L Time = 12 months	243 ± 86***	1,668 ± 496	-

### Relationship between NGAL and kidney function

**Figure 1:**
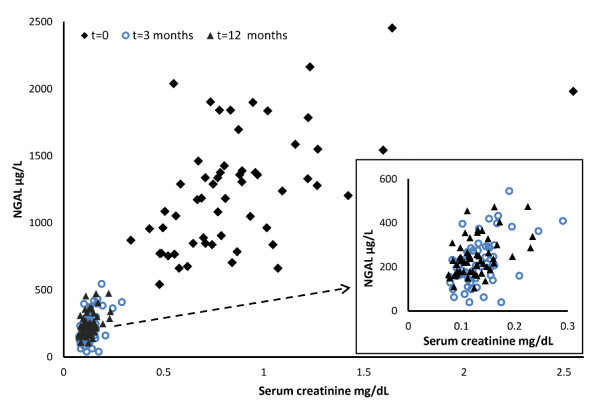
**Relationship between serum creatinine and plasma NGAL levels in the kidney allograft recipients**. Squares: before transplantation, circles: three months after transplantation, triangles: 12 months after transplantation. Insert: Three and 12 months after transplantation with expanded resolution.

Before Tx plasma NGAL concentrations were highly associated with serum creatinine (R = 0.51, p < 0.0001) and leukocyte count (R = 0.29, p < 0.026) in concordance with previously published data [21,26,37]. At three and 12 months post Tx NGAL levels were associated with homocysteine (R = 0.39, p = 0.005 and R = 0.47, p = 0.0007) and at 12 months the association with leukocyte count was mitigated reflecting the partially restored kidney function. The relationship between NGAL levels and serum creatinine is shown in Figure 1.

We found no relationship between NGAL levels and increased blood pressure defined as systolic BP > 130 mmHg and/or diastolic BP ≥ 90 mmHg in either groups both before and after transplantation. However, eGFR was significantly lower in the hypertension group after 12 months (eGFR = 71 ± 26 versus eGFR = 84 ± 18, p < 0.05).

### Recovery of recombinant NGAL

The median recovery of exogenously added recombinant NGAL in plasma was 90% for samples spiked to 3 μg/L, 97% for samples with 9 μg/L and 89% for samples with 30 μg/L. The overall median recovery was 91%. The results are shown in Table 3.

**Table 3:** Recovery of NGAL, internal replicates (n = 6), external replicates (n = 2)

NGAL level in sample	Spike μg/L	Expected μg/L	Measured μg/L	Recovery (%)
Low	3	3.8	3.8	99.9
	9	9.8	9.9	100.8
	30	30.8	27.4	89.1

Medium	3	9.8	8.1	82.9
	9	15.8	15.0	94.6
	30	36.8	31.9	86.6

High	3	11.5	9.9	86.2
	9	17.5	16.4	94.1
	30	38.5	34.8	90.6

### Comparison between platforms

**Figure 2:**
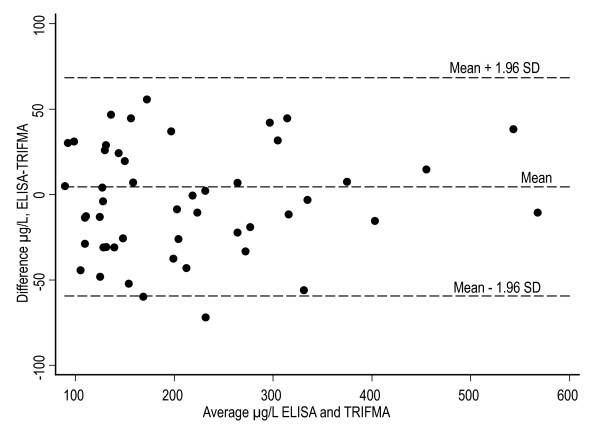
**Mean vs. absolute difference plot comparing the NGAL in-house assay with the commercial ELISA assay**. Absolute differences were calculated as values obtained using the TRIFMA assay subtracted from values obtained using the ELISA assay. The 95% limits of agreement ( ± 1.96 SD) are ± 63 μg/L. SD is the standard deviation of the difference.

**Figure 3:**
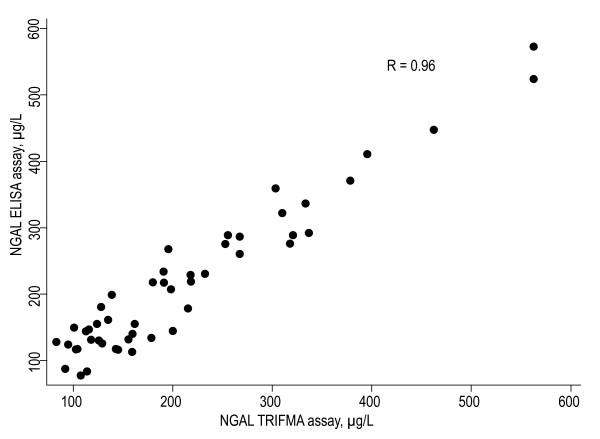
**Scatter plot; TRIFMA assay NGAL levels vs. ELISA assay NGAL levels (linear regression: R = 0.96)**.

Two samples were omitted from the analysis due to high standard deviations ( > 15%) in the in-house assay and 16 samples were out of range ( > 900 μg/L) in the ELISA assay and were omitted. These samples were re-measured, and confirmed (average CV 6%, range; 4-7%), with the in-house assay in four serial dilutions (up to 1:800) (data not shown). For the in-house assay the concentrations of NGAL ranged between 83 μg/L and 563 μg/L with mean 215 μg/L. For the ELISA, concentrations ranged between 57 μg/L and 496 μg/L with mean 186 μg/L. The two methods were compared using scatter plot, regression analysis and a Bland-Altman plot [38], Figure 2. The Bland-Altman plot shows a random distribution around the mean with a proportional difference of approximately 14% on average with higher levels reported by the TRIFMA assay. The result of a Pitman's test showed that there was no significant difference (p = 0.55) between the measuring errors of the two methods. Regression analysis, shown in Figure 3, yielded an R value of 0.96. Taken together these data indicate a high concordance between the assays.

## Discussion

This prospective study investigated the level of plasma NGAL in a cohort of kidney allograft recipients and included a uraemic control group and a group of healthy controls. The main findings of this study were that plasma NGAL levels were markedly higher in patients prior to transplantation compared to the levels measured at three and twelve months after transplantation. Plasma NGAL levels remained constant between three and twelve months but was still significantly higher than the level in the healthy control group reflecting that the kidney function is not fully restored as seen by lowered eGFR. In the Tx-group before transplantation we found that NGAL paralleled measures of kidney function and correlated to serum creatinine and leukocyte count confirming previous reports showing that elevated levels of NGAL reflect dysfunction of the kidney [21,26,37,39]. At three and twelve months after transplantation we found that high plasma NGAL levels were associated with elevated plasma homocysteine which may illustrate the atherosclerotic component of kidney disease. Patients with chronic kidney disease (CKD) (within stage 3 CKD category) have an increased risk above 20% by the Framingham risk score for developing cardiovascular disease (CVD) [40,41]. A recent study reported significant associations between plasma NGAL, plasma homocysteine, eGFR and serum creatinine in patients with advanced carotid atherosclerosis [42] highlighting the hypothesis of NGAL as a mediator of vascular remodeling and potential role in CVD [43,44]. The relevance of this association for the present study is unresolved, but raises the question of a possible relationship between homocysteine and NGAL since homocysteine is a known risk factor of CVD [45]. There was no clear relationship between NGAL and BP in the Tx- group prior to transplantation or in the uraemic group (remaining on the waiting list for one year). Several studies have reported an association between NGAL and BP. First, Eleneihoum et al. reported an association between NGAL and diastolic blood pressure in middle aged patients with early asymptomatic atherosclerosis [43]. Secondly, Bolignano et al. found association between NGAL and systolic BP in patients with congestive heart failure [43]. Thirdly, Malyszko et al. showed association of NGAL to hypertension in patients with stable coronary artery disease and normal kidney function [46] and found an association in renal allograft recipients in a cross-sectional study [24]. Finally, Giaginis et al. reported association between NGAL and hypertension in a patient group with carotid atherosclerosis [42]. In most of these studies BP was also associated to eGFR, serum creatinine or both. In the study of Giaginis et al., hypertensive patients presented with increased serum creatinine levels and reduced eGFR indicating that the association between NGAL and hypertension may be secondary to the effect of renal impairment. The reason why we did not find any association between BP, NGAL and serum creatinine, both before and three and 12 months post-Tx may be due to the limited size of our cohort. However, high BP was associated with reduced eGFR at twelve months, supporting the idea that NGAL is secondary to eGFR in terms of BP.

A limitation of our study is that we included a rather small group of patients in CKD stage 5 (eGFR < 15 ml/min), but paired longitudinal data were available to compensate for this. Also, the uraemic control group consists of older patients and with longer duration of uraemia. The use of serum creatinine and eGFR values in uraemic patients is biased and influenced by dialysis, weight and muscles of the patient and should be interpreted with caution.

## Conclusions

Plasma NGAL correlated to serum creatinine, eGFR and duration of ESRD and to serum creatinine and eGFR after transplantation. Moreover, plasma NGAL was associated with homocysteine after transplantation. It remains to be clarified whether the association between NGAL and homocysteine is caused by presently unknown factors or may be due to a further extension of the atherosclerotic component of kidney disease.

## Competing interests

The authors declare that they have no competing interests.

## Authors' contributions

NEM and AF conceived the study. NEM was responsible for the assay design, measurements of NGAL, statistical analyses and for writing the paper. MH was responsible for the statistical analyses involving clinical data. MH, KAJ, JMH, CB and BFR were responsible for the prospective study used for these analyses. All authors' contributed to the manuscript. All authors have read and approved the final manuscript.

The results presented in this paper have not been published previously in whole or part, except in abstract form.

## Pre-publication history

The pre-publication history for this paper can be accessed here:

http://www.biomedcentral.com/1471-2369/13/8/prepub
